# Feasibility and outcome of reproducible clinical interpretation of high-dimensional molecular data: a comparison of two molecular tumor boards

**DOI:** 10.1186/s12916-022-02560-5

**Published:** 2022-10-24

**Authors:** Damian T. Rieke, Till de Bortoli, Peter Horak, Mario Lamping, Manuela Benary, Ivan Jelas, Gina Rüter, Johannes Berger, Marit Zettwitz, Niklas Kagelmann, Andreas Kind, Falk Fabian, Dieter Beule, Hanno Glimm, Benedikt Brors, Albrecht Stenzinger, Stefan Fröhling, Ulrich Keilholz

**Affiliations:** 1 Comprehensive Cancer Center, Charité – Universitätsmedizin Berlin, Corporate Member of Freie Universität Berlin and Humboldt-Universität zu Berlin, Chariteplatz 1, 10117 Berlin, Germany; 2 Department of Hematology, Oncology and Cancer Immunology, Campus Benjamin Franklin, Charité – Universitätsmedizin Berlin, Corporate Member of Freie Universität Berlin and Humboldt-Universität zu Berlin, Hindenburgdamm 30, 12203 Berlin, Germany; 3 Berlin Institute of Health (BIH) at Charité – Universitätsmedizin Berlin, Anna-Louisa-Karsch-Straße 2, 10178 Berlin, Germany; 4 German Cancer Consortium (DKTK) and German Cancer Research Center (DKFZ), Heidelberg, Germany; 5 Department of Translational Medical Oncology, National Center for Tumor Diseases (NCT) Heidelberg and German Cancer Research Center (DKFZ), Heidelberg, Germany; 6 Core Unit Bioinformatics (CUBI), Berlin Institute of Health at Charité – Universitätsmedizin Berlin, Berlin, Germany; 7 Department for Translational Medical Oncology, National Center for Tumor Diseases (NCT/UCC), Dresden, Germany; 8 Faculty of Medicine and University Hospital Carl Gustav Carus, Technische Universität Dresden, Dresden, Germany; 9 Helmholtz-Zentrum Dresden - Rossendorf (HZDR), Dresden, Germany; 10 Institute of Pathology, University Hospital Heidelberg, Heidelberg, Germany

**Keywords:** Precision oncology, Whole-exome sequencing, RNA-sequencing, Clinical interpretation, Targeted therapy, Molecular tumor board

## Abstract

**Background:**

Structured and harmonized implementation of molecular tumor boards (MTB) for the clinical interpretation of molecular data presents a current challenge for precision oncology. Heterogeneity in the interpretation of molecular data was shown for patients even with a limited number of molecular alterations. Integration of high-dimensional molecular data, including RNA- (RNA-Seq) and whole-exome sequencing (WES), is expected to further complicate clinical application. To analyze challenges for MTB harmonization based on complex molecular datasets, we retrospectively compared clinical interpretation of WES and RNA-Seq data by two independent molecular tumor boards.

**Methods:**

High-dimensional molecular cancer profiling including WES and RNA-Seq was performed for patients with advanced solid tumors, no available standard therapy, ECOG performance status of 0–1, and available fresh-frozen tissue within the DKTK-MASTER Program from 2016 to 2018. Identical molecular profiling data of 40 patients were independently discussed by two molecular tumor boards (MTB) after prior annotation by specialized physicians, following independent, but similar workflows. Identified biomarkers and resulting treatment options were compared between the MTBs and patients were followed up clinically.

**Results:**

A median of 309 molecular aberrations from WES and RNA-Seq (*n* = 38) and 82 molecular aberrations from WES only (*n* = 3) were considered for clinical interpretation for 40 patients (one patient sequenced twice). A median of 3 and 2 targeted treatment options were identified per patient, respectively. Most treatment options were identified for receptor tyrosine kinase, PARP, and mTOR inhibitors, as well as immunotherapy. The mean overlap coefficient between both MTB was 66%. Highest agreement rates were observed with the interpretation of single nucleotide variants, clinical evidence levels 1 and 2, and monotherapy whereas the interpretation of gene expression changes, preclinical evidence levels 3 and 4, and combination therapy yielded lower agreement rates. Patients receiving treatment following concordant MTB recommendations had significantly longer overall survival than patients receiving treatment following discrepant recommendations or physician’s choice.

**Conclusions:**

Reproducible clinical interpretation of high-dimensional molecular data is feasible and agreement rates are encouraging, when compared to previous reports. The interpretation of molecular aberrations beyond single nucleotide variants and preclinically validated biomarkers as well as combination therapies were identified as additional difficulties for ongoing harmonization efforts.

**Supplementary Information:**

The online version contains supplementary material available at 10.1186/s12916-022-02560-5.

## Background

Precision oncology is expected to improve cancer treatment by taking into account molecular alterations [1]. Targeted treatment of well-defined molecular alterations has shown a clinical benefit, accordingly [2–4]. The precision oncology process relies on many steps, including patient accrual, sample analysis, interpretation of results, and their clinical application [5]. The clinical interpretation of molecular data from tumor sequencing has been called the “bottleneck” of precision oncology [6]. Published guidelines address variant annotation and biomarker prioritization, whereas a complete interpretation workflow remains unstandardized [7–11]. Multiple databases and search tools exist for the identification of biomedical literature to support biomarker associations [12–14]. Yet, most databases contain non-overlapping literature [15, 16]. The vast biomedical literature and challenges in the variant interpretation process lead to inter-interpreter differences even with limited molecular data [17]. The use of multi-gene panels to simultaneously interrogate multiple genes of interest has become a standard in most cancer centers. In addition to gene-panel diagnostics, even more comprehensive analyses of genome or transcriptome are increasingly used [18–21], thus further raising dimensionality and therefore complexity of the resulting data. These analyses hold promise to identify targetable alterations in patients where no well-defined biomarker will be identified by more targeted analyses. For unselected patient cohorts, a clear benefit with precision oncology has so far not been shown in prospective studies [22–24]. These results contrast with the clear benefit of precision oncology strategies in patients with well-defined molecular alterations within a specific tumor histology [3, 4]. A few more recent trials have shown efficacy of biomarker-directed therapy even in histology-agnostic trials [2, 25]. In order to further expand these benefits to a larger and unselected patient population, reproducible and evidence-based strategies for the clinical interpretation of complex molecular data are required. In order to identify challenges for harmonized workflows, we compared treatment options identified by two independent molecular tumor boards for patients with identical exome and transcriptome sequencing, e.g., high-dimensional, molecular data.

## Results

### Analysis of MTB workflows

The workflows of both MTB were compared (Additional file 1, Fig S1). Both workflows consisted of initial steps of biomarker identification and filtering from the provided list of molecular alterations, followed by clinical annotation and molecular tumor board discussion, treatment recommendation, and clinical follow-up. Importantly, both workflows included structured literature searches and the use of databases, evidence levels, and interdisciplinary discussion of results. Differences were observed with an additional step of validation for the analysis of missing data and the interpretation of biomarkers in their genomic context in the Berlin workflow and a step for the functional assessment of molecular changes using literature and database searches in the Heidelberg workflow. However, both additional steps were largely included in the respective other workflows (validation under clinical relevance in the Heidelberg workflow and functional assessment under filtering in the Berlin workflow).

### Patient characteristics

**Fig. 1:**
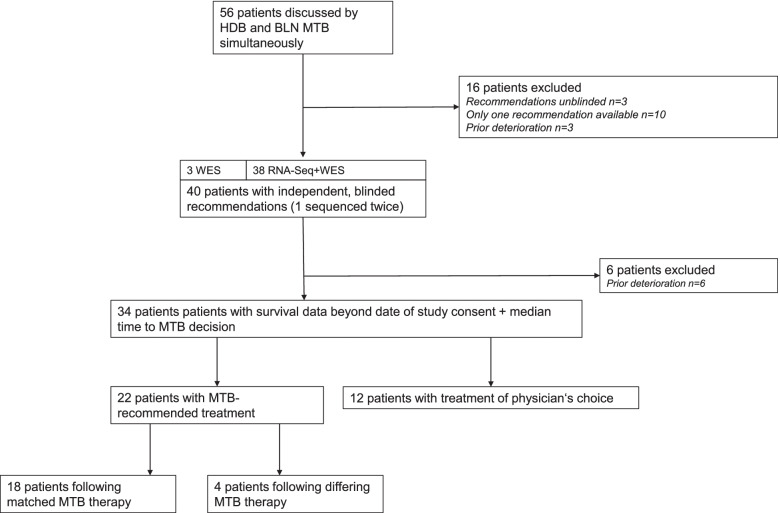
Consort diagram depicting the flow of patients

We identified 56 patients who were discussed independently by MTBs in Heidelberg and Berlin between 2016 and 2018. Sixteen patients were excluded from the analysis because of prior deterioration, only one available treatment recommendation, or identical molecular information was not available to one of the tumor boards at the time of discussion or the tumor board was not blinded to results from the other MTB (Fig. 1). Clinical characteristics of the resulting 40 patients are displayed in Table 1. The median age at the date of trial inclusion was 45 years. Slightly more male than female patients were amongst the study participants. All patients received prior systemic chemotherapy before DKTK MASTER inclusion. Head and neck (35%) and gastrointestinal tumors (30 %) were the most frequently sequenced tumor sites. Neuroendocrine neoplasm G3 was the most common histology.

**Table 1:** The clinical data of the 40 patients included in the analysis

Clinical characteristics	Value
**Age, years**	
Median (range)	45 (22–65)
**Sex, no. (%)**	
Male	22 (55)
Female	18 (45)
**Prior systemic chemotherapies, no. (%)**	
1	5 (12.5)
2	12 (30)
3	8 (20)
≥ 4	15 (37.5)
**Tumor site/type, no. (%)**	
**Head and neck**	**14 (35)**
Neuroendocrine neoplasm G3	4
Squamous cell carcinoma	3
Adenoid cystic carcinoma	3
Adenocarcinoma	2
Chondrosarcoma	1
Carcinosarcoma	1
**Gastrointestinal**	**12 (30)**
Neuroendocrine neoplasm G3	6
Neuroendocrine MANEC	2
Neuroendocrine neoplasm G2	1
GIST	1
Mesothelioma	1
Squamous cell carcinoma	1
**Urogenital**	**8 (20)**
Neuroendocrine neoplasm G3	2
Germ cell tumor	2
Leiomyosarcoma	1
Pheochromocytoma	1
Teratoma	1
Squamous cell carcinoma	1
**Lung**	**3 (7.5)**
Adenocarcinoma	2
Neuroendocrine neoplasm G2	1
**Cancer of unknown primary**	**3 (7.5)**
Adenocarcinoma	1
Neuroendocrine neoplasm G3	1
DSRCT	1

*GIST* gastrointestinal stromal tumor, *MANEC* mixed adenoceuroendocrine carcinoma, *DSRCT* desmoplastic small round cell tumor

**Fig. 2:**
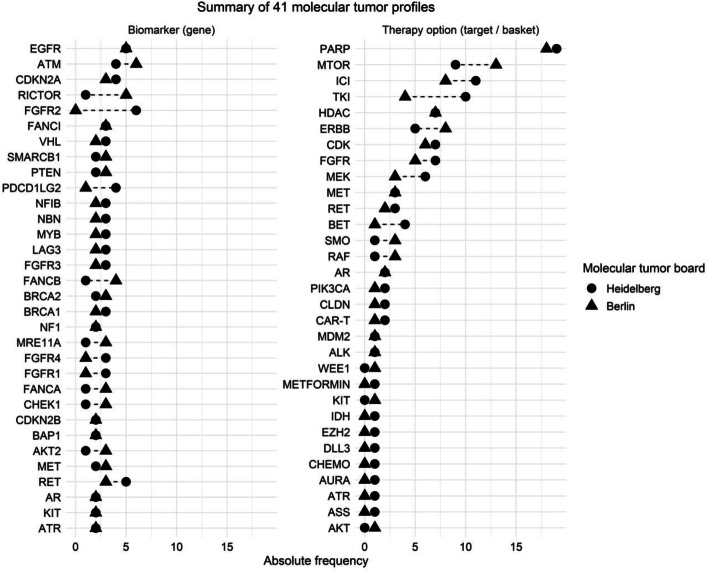
Summary of biomarker genes and therapy options from 41 molecular tumor profiles. The left panel indicates genes that were identified at least four or more times by molecular tumor boards in Heidelberg (dot) and Berlin (triangle) as biomarkers. The right panel shows all recommended therapy options by both MTB. Abbreviations of therapy options, are as follows: ICI, immune checkpoint inhibition; TKI, tyrosine kinase inhibitors; CLDN, claudin-directed therapy; CAR-T, chimeric antigen receptor T-cell; CHEMO, chemotherapy; ASS, acetylsalicylic acid; all other gene names refer to treatment directed against these targets

WES and RNA-Seq were performed on 38 samples of 37 patients, and WES only on 3 samples of 3 patients, since tumor material was insufficient for RNA-Seq. One patient was successfully sequenced a second time after progression and both sequencing results were independently considered for analysis. A median of 309 (WES and RNA-Seq) and 82 (WES) aberrations per patient were reported to the MTB, of which the majority were gene expression outliers in the WES/RNA-Seq group and SNV in the WES group (Table 2). A summary of genes and respective alterations can be found in Additional file 2: Table S1. These genetic alterations then underwent annotation and interdisciplinary discussion in the MTBs in Heidelberg and Berlin. From these bioinformatically generated lists of all molecular alterations, clinical annotation identified a median of 4 predictive biomarkers per patient in both MTB. Gene expression outliers and structural alterations (e.g., gene deletion/amplification) were the molecular alteration types that were most commonly identified as predictive biomarkers. These findings resulted in a median of 3 and 2 treatment options per patient, respectively (Table 3). Predictive biomarkers and treatment options are provided in Additional file 2: Table S2. The most frequently identified alterations that were considered predictive biomarkers were aberrations of the EGFR, ATM, and CDKN2A genes (Fig. 2). Most frequently identified treatment options included PARP inhibitors, followed by mTOR and immune checkpoint (ICI) inhibitors, as well as various receptor tyrosine kinase (TKI) inhibitors (including multi-kinase, FGFR, and ERBB inhibitors) (Fig. 2).

**Table 2:** Median number and range of all molecular alterations that were reported to both MTB for clinical interpretation in the WES and RNA-Seq and WES only groups

Molecular characteristics	WES + RNA-seq ***n*** = 38	WES ***n*** = 3
Aberrations, no.		
Median (range)	309 (181–6013)	82 (24–9248)
Single nucleotide variants, no.		
Somatic median (range)	48.5 (2–5645)	73 (22–9139)
Germline median (range)	1 (0–5)	1 (0–3)
Indels, no.		
Somatic median (range)	3 (0–21)	6 (2–107)
Germline median (range)	0 (0–2)	0 (0–1)
Structural variants, no.		
Median (range)	99.5 (5–731)	61 (53–426)
Gene fusions, no.		
Median (range)	83 (0–663)	N/A
Gene expression outlier, no.		
Median (range)	166 (105–393)	N/A

**Table 3:** The median number of biomarkers and treatment options, as identified by Heidelberg and Berlin MTBs, respectively. The median number of types of alterations is also provided for biomarkers. Each predictive biomarker was counted once, irrespective of the number of resulting treatment options

Interpretations	Heidelberg	Berlin
Biomarker, no.		
Median (range)	4 (2–14)	4 (1–21)
Single nucleotide variants	1 (0–6)	1 (0–4)
Structural variants	1 (0–5)	1 (0–13)
Gene fusions	0 (0–1)	0 (0–1)
Gene expression outlier	2 (0–7)	1 (0–5)
Signatures	0 (0–3)	0 (0–1)
Treatment options, no.		
Median (range)	3 (1–5)	2 (1–6)

### Treatment recommendation and predictive biomarker agreement

Identified treatment options were compared between both MTBs. The mean overlap coefficient between both molecular tumor boards was 66%. The respective treatment options for the analyzed cohort are provided in Additional file 2: Table S3.

**Fig. 3:**
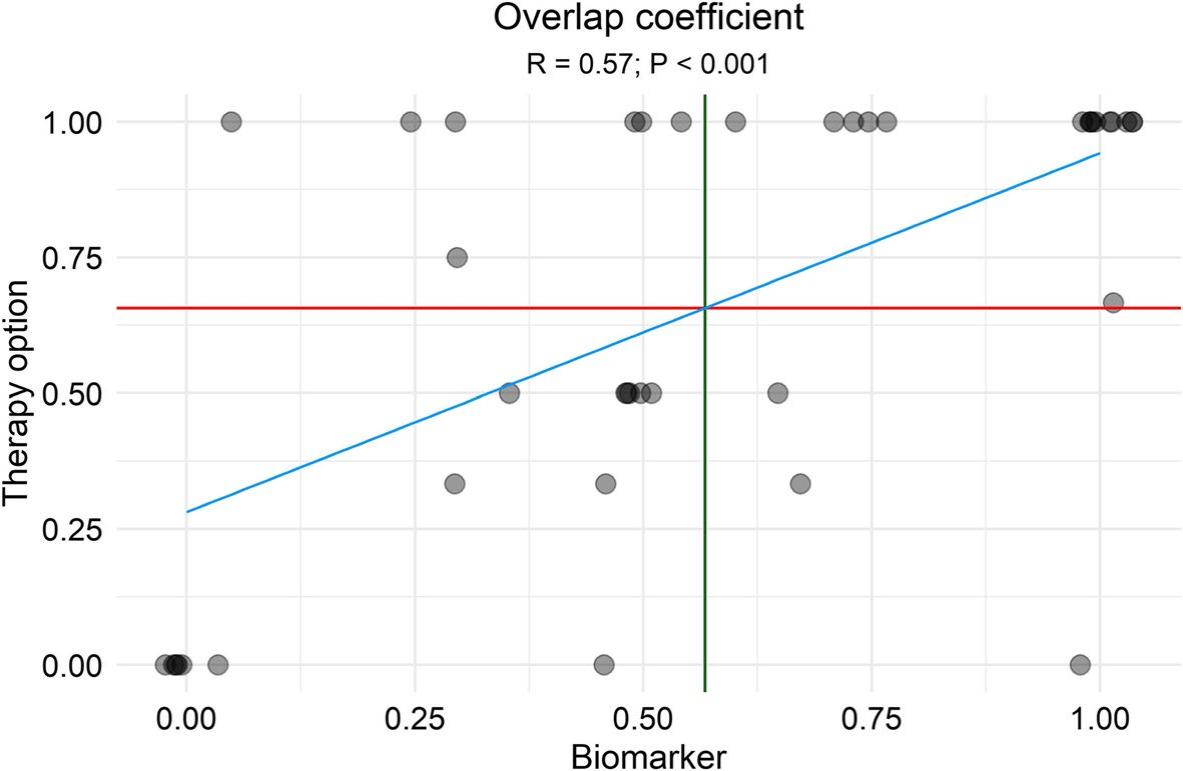
Analysis of an association between concordant biomarkers (*x*-axis) and therapy options (*y*-axis) between both molecular tumor boards. The plot demonstrates a significant positive correlation (blue line, *R* = 0.57; *P* < 0.001) between biomarkers and therapy options identified from 41 unique molecular tumor profiles (each represented by a dot). The red line represents the mean overlap coefficients for therapy options, and the green line for biomarkers

There was a significant positive correlation between the identification of identical predictive biomarkers and identification of identical treatment recommendations between both MTBs (*R* 0.57, *p* < 0.001, Fig. 3).

**Fig. 4:**
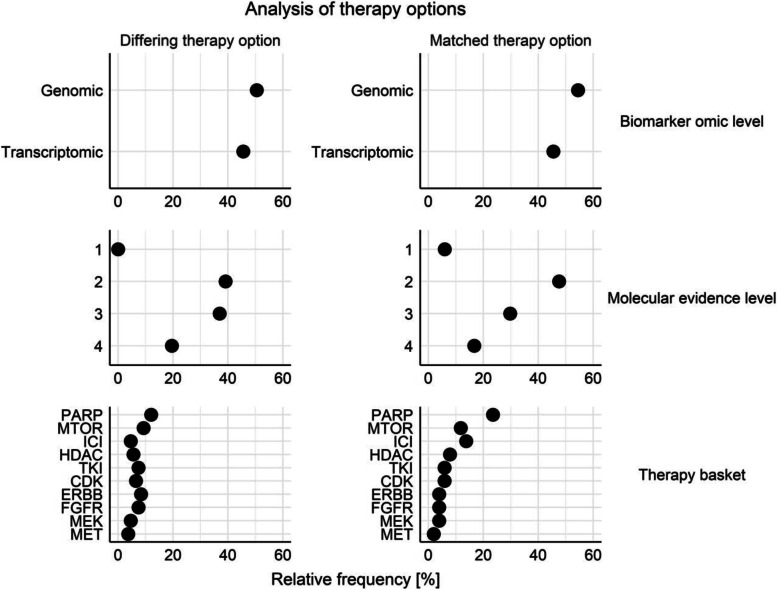
Analysis of differing and matched therapy options between molecular tumor boards. The left-sided panel demonstrates the relative frequency of differing therapy options according to the molecular level of supporting biomarkers, the underlying molecular evidence level, and the ten most frequent therapeutic baskets. The corresponding relative frequencies for matched therapy options are shown in the right panel

To identify challenges for future harmonization efforts, treatment options were analyzed with regard to the type of biomarker, the evidence level (EvL), and therapeutic basket (Fig. 4). More concordant recommendations were seen for genomic compared to transcriptomic biomarkers, with clinical (EvL 1 and 2) compared to preclinical (EvL 3 and 4) evidence levels and immune checkpoint and PARP inhibitors compared to mTOR inhibitors. A mean of 1.7 predictive biomarkers were reported per treatment option. No significant association was seen between the number of reported supporting predictive biomarkers and concordance of treatment options, although more predictive biomarkers (mean 1.8 vs. 1.5, *t*-test *p*=0.06) were reported per concordant treatment option than per discordant treatment option. Recommended combination therapies were assessed individually and only one of 25 recommended combination regimens was identically recommended by both MTB.

### Analysis of clinical impact

**Fig. 5:**
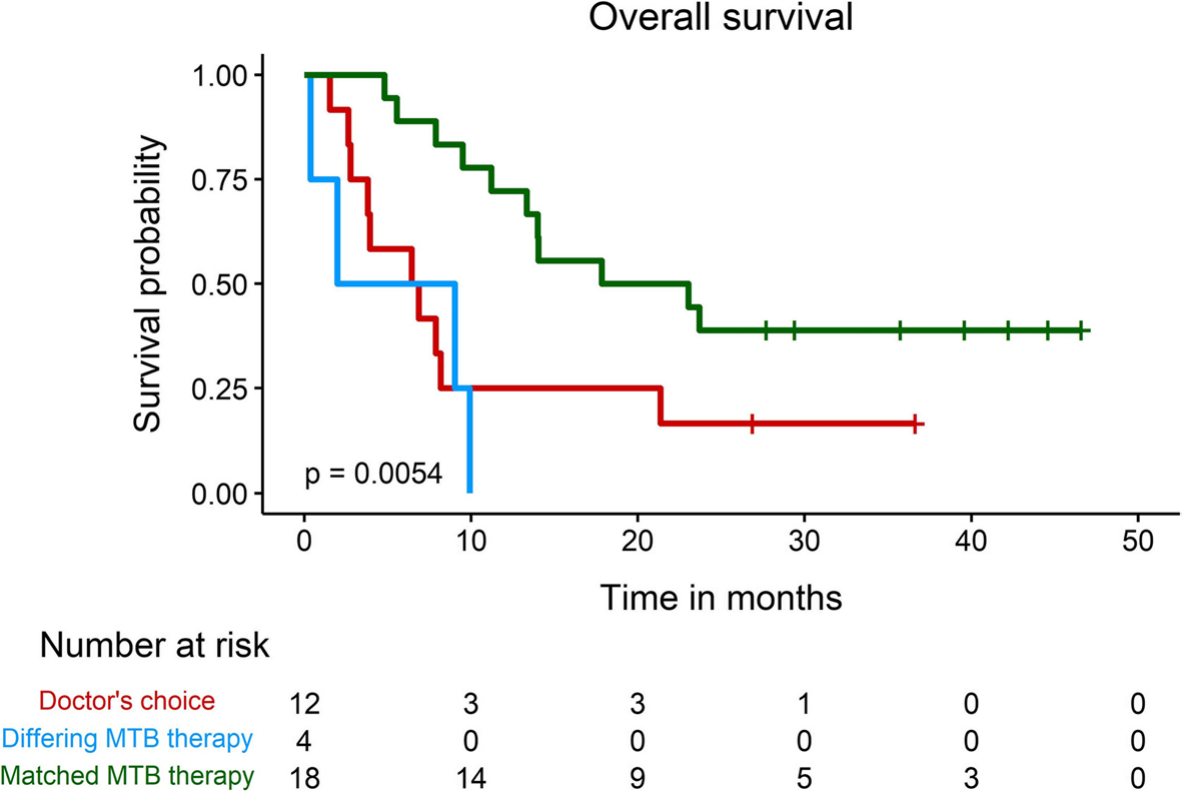
Clinical outcome. The Kaplan-Meier survival curve of 34 patients eligible for overall survival analysis revealed a significantly improved outcome for patients stratified to matched MTB therapy options (e.g., treatment recommended by both MTB), compared with those receiving treatment based on differing MTB recommendations (e.g., treatment recommended by only one MTB) or doctor’s choice (e.g., treatment not recommended by any MTB)

After communication of identified treatment options, 22 of 40 (55 %) patients started at least one recommended therapy. Eighteen out of these 22 patients received treatment following concordant treatment recommendations (Figs. 1 and 5).

Patients receiving therapy as recommended by both MTBs (Matched MTB therapy) displayed a significantly longer overall survival (*P* = 0.005, Fig. 5), compared to the groups of patients whose initiated therapy followed a treatment option that was suggested by only one MTB (Differing MTB therapy) or physician’s choice (e.g., therapy not recommended by any MTB).

## Discussion

Specific attention and additional research is required to improve the clinical annotation of molecular data, which is still unstandardized and inconsistent between molecular tumor boards [17]. The integration of high-dimensional molecular data can be expected to further complicate clinical interpretation but no real-world data currently exist on the scale or clinical impact of this scenario. Alleviating this imminent “bottleneck” [6] is expected to improve clinical decision-making and the prospective design of clinical trials for precision oncology.

In this work, we retrospectively analyzed the clinical interpretation of identical and high-dimensional molecular alterations of 40 patients by two molecular tumor boards that were prospectively sequenced within the DKTK-MASTER-program. This analysis yielded a mean overlap coefficient of 66%. Previous studies of recommendation heterogeneity yielded overall agreement rates between 40% [17] and 86% [26]. However, major differences between the studies have to be taken into account, when comparing these data. The average number of molecular alterations per patient was 8 in the study by Rieke et al. and 2.6 in the study by Koopman et al. In the here presented study, more than 300 alterations per patient, identified by whole-exome and RNA-sequencing, were clinically interpreted. Furthermore, the study by Koopman et al. assessed clinical interpretation in well-defined clinical situations of melanoma and NSCLC samples. The DKTK-MASTER study was designed to include patients without established treatment options, which is highlighted by the large number of patients with neuroendocrine neoplasms in this cohort, for which no standard of treatment exists in later lines of systemic therapy. Considering these molecular and clinical challenges in an unplanned retrospective analysis of an experimental sequencing study, an overall agreement rate of more than 60% should be viewed as highly encouraging. These results could be mediated by similar MTB workflows, with an interdisciplinary MTB discussion after prior manual annotation of molecular aberrations with evidence levels, following a structured search of databases. Further improvement could therefore be expected with ongoing harmonization efforts.

This analysis allows for a detailed look at challenges with the interpretation of complex molecular data for these efforts. Generally, more heterogeneous recommendations were found in the setting of biomarkers with low evidence levels and combination therapy, probably due to the wealth and heterogeneity of preclinical studies [15], differences in their appreciation [11], and a lack of controlled trials for combination targeted therapy due to combinatorial complexity [27]. More data for the rational use of drug combinations for precision oncology is warranted. Additionally, lower agreement rates were identified for gene expression data. This is probably caused by a lack of clinical studies for most of these alterations, which are additionally not well-defined because of a lack of established cut-offs. Despite these more obvious challenges, perfect matches were also not achieved for SNV. Most SNVs annotated in this cohort were not identified in well-characterized genes and therefore created challenges in their appreciation as potential biomarkers.

The agreement rate in our study was lower for identified predictive biomarkers than the corresponding treatment option. This can be explained by the frequent identification of several alterations that point towards identical treatment recommendations (e.g., BRCA mutation, ATM underexpression, FANCI, FANCA deletion for PARP inhibitors in the same patient) but were not always all named by both tumor boards. Yet, agreement rates of predictive biomarkers were significantly associated with agreement rates of treatment options. Therefore, the structured identification of potential (predictive) biomarkers from molecular data remains key to the identification of treatment options. Efforts for a harmonization of databases is expected to greatly aid with this [16]. Interdisciplinary teams will be increasingly important to extract the maximum of clinically relevant data from complex molecular profiles.

Some limitations of this study should be considered: Patients were discussed in parallel by the molecular tumor boards in Heidelberg and Berlin only in the beginning of the MASTER program, thus limiting the number of patients. Additionally, the recommendations reflect MTB practices of the inclusion years 2016–2018, which have evolved significantly since then, thus possibly underestimating current concordance rates. This limitation is important, since great efforts have been put into biomarker annotation and database development, and since efforts are ongoing [16, 28]. A follow-up analysis of the same patient data was not feasible within a blinded MTB setting. However, a more superficial second visit of the data suggested higher agreement rates between participating tumor boards.

The availability of new data and treatment options will eventually bring established targets into clinical routine and away from a more experimental MTB setting. An analysis of unclear and complex data can therefore be expected to remain a problem for precision oncology. Therefore, this analysis also has several strengths: Identical molecular data were available, limiting the effect of potential confounders. The retrospective, real-world design of this study reduced the risk of bias. Furthermore, the interpreted molecular data are highly complex, incorporating WES and RNA-Seq data, and thus allow for an analysis of a wealth of biomarkers and treatment recommendations even in the setting of two participating MTBs. High-dimensional molecular data beyond targeted sequencing are increasingly incorporated into precision oncology [18–21]. The added value of whole-exome/genome and RNA-sequencing data is an important clinical question. In our cohort, 75–90% of 67 SNV or Indels that were used as predictive biomarkers would have been identified with large multigene-panels (e.g., MSK-IMPACT, TSO500). Yet, most structural/transcriptomic biomarkers that led to treatment recommendations or further supported them would not have been identified and the patient number is too small for a definitive analysis of the clinical impact. Therefore, this question should be answered in larger and ideally prospective trials. In the WINTHER trial, no improvement of outcome but a numerically better clinical benefit ratio could be shown with transcriptomic profiling [18].

An improved outcome could be shown in the I-PREDICT and WINTHER trials, as well as a real-world data analysis, for patients that received treatment that was better matched to their tumor’s molecular profiles [18, 23, 29]. In our retrospective analysis in a small cohort of patients, concordant treatment recommendations were also associated with an improved overall survival. Since patients with more well-defined molecular alterations were more likely to receive reproducible recommendations and effective treatment, this finding might reflect that patients receiving treatment that is well-matched to their unique molecular tumor profile achieve a greater clinical benefit—thus mirroring the results from the I-PREDICT and WINTHER trials in a setting with complex molecular data. Yet, this analysis should be viewed with caution given the low number of patients, retrospective analysis, and selection bias. Prospective trials with a focus on interpretation practices (such as matching scores and reproducibility of treatment options) are warranted to validate these exploratory findings.

## Conclusion

Reproducible and evidence-based interpretation of complex molecular data is feasible with the use of structured workflows. Additional attention is required for the interpretation of data beyond genomic analyses and biomarkers with preclinical evidence levels as well as for the introduction of rational combination therapies.

## Methods

### Patient recruitment

Patients with advanced solid tumors of a rare histology or younger age (< 50y), no available standard therapy, and available fresh-frozen tumor tissue were included in the DKTK-MASTER precision oncology program of the German Cancer Consortium (DKTK) [21]. Patients with molecular profiles that were independently discussed by Heidelberg and Berlin molecular tumor boards were considered eligible for this analysis and included into the MASTER program between 2016 and 2018. The study was approved by the local ethics committees (Heidelberg, Berlin). Written, informed consent was obtained from all participants before inclusion into the study.

### Sequencing and variant identification

Fresh-frozen tumor tissue was obtained from all participating patients and shipped to the central laboratory in Heidelberg. Specimen handling, DNA and RNA extraction, next-generation sequencing, and bioinformatic analyses were performed as published [21]. Briefly, DNA and RNA from tumor specimen and DNA from matched blood samples were isolated using the AllPrep Mini or Universal Kits (Qiagen). After library preparation (SureSelect Human All Exon, Agilent; TruSeq RNA Sample Preparation kit V2, Illumina), whole-exome and RNA-paired-end sequencing (2 × 151 bp; 2 × 101 bp) was performed with various HiSeq instruments (e.g., HiSeq 2000, 2500, and 4000; Illumina). Reads were aligned and mapped and single nucleotide variants (SNV)/indel/structural variant and copy number alterations were analyzed. An integrated file was generated per patient and annotated using dbNSFP version 2.9 (http://varianttools.sourceforge.net/Annotation/dbNSFP), using functional impact tools as well as several gene lists of interest, including the Cancer Gene Census (https://cancer.sanger.ac.uk/census). One excel file was generated per patient including single nucleotide variations/indels, gene fusions and structural variations, mutational signatures, RNA information, and germline findings, if available. RNA expression ranking was based on the first 148 RNA samples of the MASTER program. These information were provided to the molecular tumor boards.

### Molecular tumor board workflows

Molecular tumor boards (MTB) were established independently in Heidelberg and Berlin. In Berlin, a weekly meeting comprising at least medical oncologists, bioinformaticians, pathologists, and molecular biologists was established. In Heidelberg, a MTB was held in parallel, with medical oncologists, bioinformaticians, pathologists, and molecular biologists in regular weekly attendance. Prior to the MTB, specialized physicians analyzed the identified alterations (e.g., SNV/indels, gene expression alterations, gene fusions, structural variations, mutational signatures) using at least one database as well as a structured PubMed search to identify potential biomarkers. After establishment of a list of potential biomarkers, predictive information were collected and annotated using predefined evidence levels [11, 20]. Predictive biomarkers were defined as molecular alterations providing information on the probability of a response to a particular therapy [30]. For this analysis, only predictive biomarkers identified within the MASTER program (e.g., WES/RNA-Seq) were considered. Predictive information were collected from structured literature and database searches (e.g., PubMed, CIViC, OncoKB) including clinical and preclinical data [12, 13]. Identified predictive information on the respective alterations was summarized and sent to members of the MTB prior to the meeting. Predictive biomarkers were then discussed in the MTB to identify treatment options, taking into account the evidence level, existence of multiple biomarkers for the same drug, and patient characteristics. These were communicated to the treating physician. The workflow has been published previously [20] (Additional File 1: Fig. S1). Regular, standardized follow-up was performed to analyze treatment outcomes of patients after MTB discussion [21].

### Data collection and analysis

Patients with treatment recommendations from an independent discussion of identical molecular results in both MTBs were identified from the internal MTB database. Number and type of molecular alterations were collected from the initial report as provided to both molecular tumor boards. Identified biomarkers and treatment options were retrieved from the final MTB recommendations and supporting documents used in the MTB discussion. Survival data were retrieved from follow-up documents. Data were then structured in a harmonized format and analyzed using R (R version 4.0.0 (2020-04-24) -- “Arbor Day”. R: A language and environment for statistical computing. R Foundation for Statistical Computing, Vienna, Austria. URL http://www.R-project.org/).

Treatment recommendations were defined as treatment options that were identified and communicated after multidisciplinary discussion in the respective MTB (e.g., immune checkpoint inhibition). Predictive biomarkers were defined as genetic aberrations that were used for the identification of treatment recommendations after annotation and multidisciplinary discussion in the MTB, thus allowing for multiple predictive biomarkers as a rationale for one identified treatment option (e.g., high tumor mutational burden and POLE mutation as predictive biomarkers for immune checkpoint inhibition as treatment recommendation).

Treatment recommendations and predictive biomarkers were matched for each individual patient. The overlap coefficient was calculated using the Szymkiewicz–Simpson formula $overlap\left(X,Y\right)=\frac{\left|X\cap Y\right|}{\min \left(\left|X\right|,\left|Y\right|\right)}$ [31].

## Supplementary Information

**Additional file 1: Figure S1.** Shows the respective molecular tumor board workflows of Heidelberg (HDB) and Berlin (BLN). Corresponding steps are indicated by their respective colors.

**Additional file 2: Table S1.** Reports all gene names and corresponding number of alterations, including SNV, gene expression outliers, indels and gene fusions that were identified in the cohort. **Table S2**. Lists the genetic alterations that were identified as predictive biomarkers and corresponding treatment options per patient by Heidelberg (HD) and Berlin (BLN) MTBs. Results from a second sequencing for the same patient are indicated by a comma. **Table S3.** Provides information on treatment options and respective biomarkers for the analyzed cohort. Results from a second sequencing for the same patient are indicated by a comma.

## Data Availability

The datasets supporting the conclusions of this article are included within the article and its additional files.

## Abbreviations

ASS: Acetylsalicylic acid
ATM: Ataxia telangiectasia mutated
BRCA: Breast cancer susceptibility protein
CAR-T: Chimeric antigen receptor T-cell
CHEMO: Chemotherapy
CLDN: Claudin-directed therapy
CTLA-4: Cytotoxic T-lymphocyte-associated protein 4
DKTK: Deutsches Konsortium für translationale Krebsforschung (German cancer consortium)
DSRCT: Desmoplastic small round cell tumor
ECOG: Eastern Cooperative Oncology Group
EvL: Evidence level
FANCA: Fanconi anemia, complementation group A
FANCI: Fanconi anemia, complementation group I
GIST: Gastrointestinal stromal tumor
HR: Hazard ratio
ICI: Immune checkpoint inhibition
I-PREDICT: Investigation of Profile-Related Evidence Determining Individualized Cancer Therapy
MANEC: Mixed adenoceuroendocrine carcinoma
MASTER: Molecularly Aided Stratification for Tumor Eradication
MTB: Molecular tumor board
OS: Overall survival
PD-1: Programmed cell death protein 1
RNA-Seq: RNA sequencing
SNV: Single nucleotide variant
TKI: Tyrosine kinase inhibitor
WES: Whole-exome sequencing
WINTHER: A Study to Select Rational Therapeutics Based on the Analysis of Tumor and Matched Normal Tissue Biopsies in Subjects with Advanced Malignancies
