# Supplementary material for: Feasibility and outcome of reproducible clinical interpretation of high-dimensional molecular data: a comparison of two molecular tumor boards
Source: BMC Med. 2022 Oct 24;20:367. doi: 10.1186/s12916-022-02560-5 (PMC9590222; doi:10.1186/s12916-022-02560-5)
Supplement: Supplementary file 1 — Additional file 1: Figure S1. Shows the respective molecular tumor board workflows of Heidelberg (HDB) and Berlin (BLN). Corresponding steps are indicated by their respective colors. [file 12916_2022_2560_MOESM1_ESM.pptx]

## Slide 1
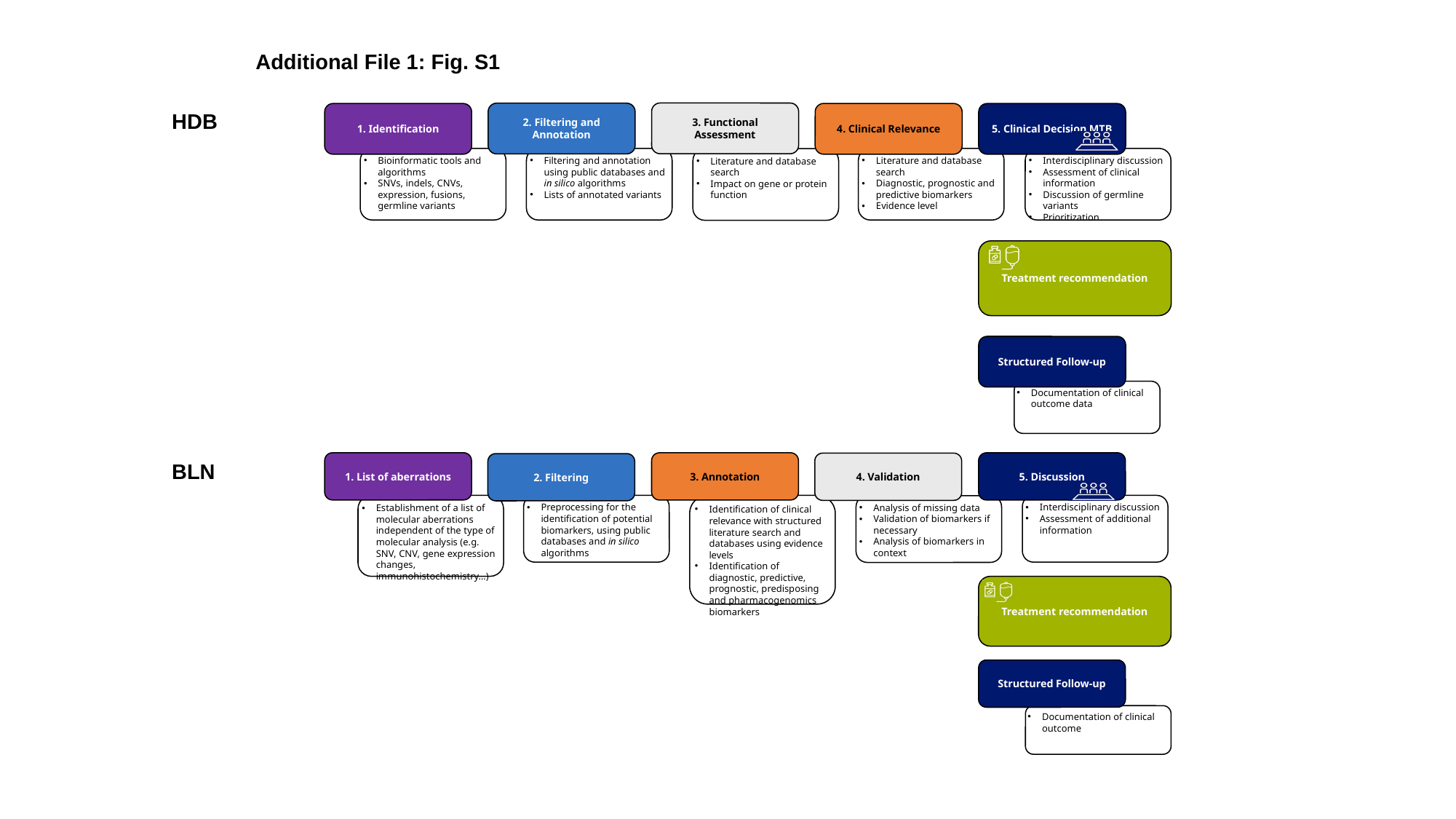

Additional File 1: Fig. S1
HDB
3. Functional Assessment
2. Filtering and Annotation
1. Identification
4. Clinical Relevance
5. Clinical Decision MTB
Bioinformatic tools and algorithms
SNVs, indels, CNVs, expression, fusions, germline variants
Filtering and annotation using public databases and in silico algorithms
Lists of annotated variants
Literature and database search
Diagnostic, prognostic and predictive biomarkers
Evidence level
Interdisciplinary discussion
Assessment of clinical information
Discussion of germline variants
Prioritization
Literature and database search
Impact on gene or protein function
Treatment recommendation
Structured Follow-up
Documentation of clinical outcome data
BLN
1. List of aberrations
3. Annotation
5. Discussion
4. Validation
2. Filtering
Preprocessing for the identification of potential biomarkers, using public databases and in silico algorithms
Interdisciplinary discussion
Assessment of additional information
Identification of clinical relevance with structured literature search and databases using evidence levels
Identification of diagnostic, predictive, prognostic, predisposing and pharmacogenomics biomarkers
Analysis of missing data
Validation of biomarkers if necessary
Analysis of biomarkers in context
Treatment recommendation
Structured Follow-up
Documentation of clinical outcome
Establishment of a list of molecular aberrations independent of the type of molecular analysis (e.g. SNV, CNV, gene expression changes, immunohistochemistry…)
